# De-crystallization of Uric Acid Crystals in Synovial Fluid Using Gold Colloids and Microwave Heating

**DOI:** 10.5101/nbe.v6i4.p104-110

**Published:** 2014

**Authors:** Bridgit Kioko, Taiwo Ogundolie, Morenike Adebiyi, Yehnara Ettinoffe, Caleb Rhodes, Brittney Gordon, Nishone Thompson, Muzaffer Mohammed, Biebele Abel, Kadir Aslan

**Affiliations:** Morgan State University, Department of Chemistry, 1700 East Cold Spring Lane

**Keywords:** Microwave-induced temperature gradients, Uric acid, Hyperuricemia, Metal assisted and microwave accelerated evaporative crystallization, Aold colloids, Microwave heating

## Abstract

In this study, we demonstrated a unique application of our Metal-Assisted and Microwave-Accelerated Evaporative Crystallization (MA-MAEC) technique for the de-crystallization of uric acid crystals, which causes gout in humans when monosodium urate crystals accumulate in the synovial fluid found in the joints of bones. Given the shortcomings of the existing treatments for gout, we investigated whether the MA-MAEC technique can offer an alternative solution to the treatment of gout. Our technique is based on the use of metal nanoparticles (i.e., gold colloids) with low microwave heating to accelerate the de-crystallization process. In this regard, we employed a two-step process; (i) crystallization of uric acid on glass slides, which act as a solid platform to mimic a bone, (ii) de-crystallization of uric acid crystals on glass slides with the addition of gold colloids and low power microwave heating, which act as “nano-bullets” when microwave heated in a solution. We observed that the size and number of the uric acid crystals were reduced by >60% within 10 minutes of low power microwave heating. In addition, the use of gold colloids without microwave heating (i.e. control experiment) did not result in the de-crystallization of the uric acid crystals, which proves the utility of our MA-MAEC technique in the de-crystallization of uric acid.

## Introduction

Gout disease, which is also known as hyperuricemia, occurs when uric acid (i.e., monosodium urate) crystals are formed in the joints of bone due to the accumulation of uric acid present in the blood. The crystallization of uric acid occurs due to the following reasons: (i) when the body increases the amount of uric acid it produces, (ii) the kidneys fail to excrete enough uric acid, and (iii) dietary habits, such as, consumption of food with high purine content and/or excessive alcoholic beverages, when a person has had an organ transplant, being overweight, or weak metabolism to break down purines. Gout is known to affect the *hallux rigidus* of the big toe with the symptoms of redness, stiffness and swelling of the big toe. In addition, other parts of the body (ankles, heels, knees, wrists, fingers, and elbows) can also be affected.

There are several reported drugs for the treatment of gout, which include anti-inflammatory drugs (NSAIDs) [1], allopurinol [2], colchicine [3], and uricosuric agents [4]. NSAIDs are prescribed to patients because of their ability to reduce inflammation in the affected areas. Despite widespread use of the drugs for the treatment gout, their side-effects, such as stomach bleeding and ulcers, thinning bones, poor wound healing and decreased ability to fight infection pose a threat to human health. In this regard, there is still an urgent need for new methodologies for the treatment of gout while minimizing the risks of other bodily complications.

Recently, the Aslan Research Group has developed a technique called Metal Assisted and Microwave Accelerated Evaporative Crystallization (MA-MAEC), in which organic and drug compounds achieve complete crystal growth in a fraction of the time when compared to conventional techniques [5-8]. The MA-MAEC technique is based on combined use of metal nanoparticles that are immobilized to solid surfaces and microwave heating, where a microwave-induced temperature gradient is created between the metal nanoparticles (cooler) and the solution (warmer) during microwave heating. As a result of microwave-induced temperature gradient, the drug compounds are forced to move from the warmer solution to the surface of cooler metal nanoparticles, where nucleation and crystallization processes occur.

**Scheme 1:**
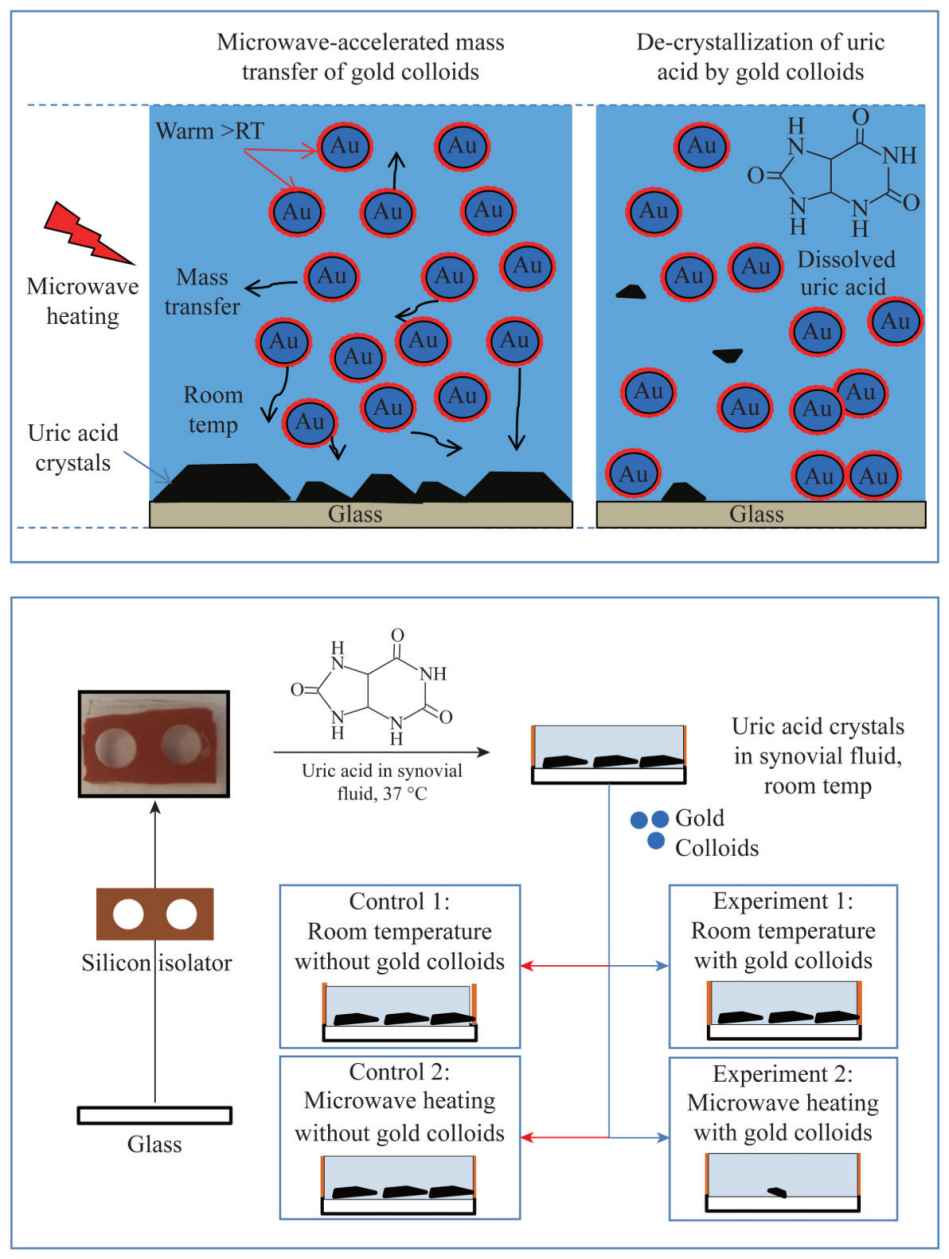
(Top) Shows the depiction of the de-crystallization of uric acid crystals with gold colloids and control sample (without gold colloids). (Bottom) Experimental procedures used in this study.

One can modify the MA-MAEC technique by the use of metal colloids in solution and immobilization of the crystals on to the solid surfaces (Scheme 1-Top), where microwave-induced temperature gradient still exists. In this regard, metal colloids in solution are used for de-crystallization of uric acid crystals. Metal colloids in solution convert the microwave energy to kinetic energy to move about the uric acid solution for de-crystallization process, where the collisions between the metal colloids and uric acid result in the break down uric acid crystals (Scheme 1-Top).

In this communication, we explore the use of gold colloids using our MA-MAEC technique for de-crystallization of uric acid. This was performed on a blank modified glass slide as our platform, where uric acid was crystallized and de-crystallized with the addition of gold colloids. The combined use of gold colloids and microwave heating resulted in the de-crystallization of uric acid crystals (i.e., 60% reduction in number of uric acid crystals). On the other hand, the use of microwave heating and gold colloids separately or at room temperature experiments did not result in the de-crystallization of uric acid crystals, which proves the effectiveness of using gold colloids and microwave heating together.

## Methods and Materials

### Materials

Sulfuric acid and hydrogen peroxide purchased from Pharmco products Inc. Deionized water purified via a Millipore Direct Q 3 UV apparatus. Glass slides of 0.96 to 1.06 mm thickness purchased from Corning Incorporated. Uric acid and 20 nm gold colloids purchased from Sigma-Aldrich (USA, catalog number: 741965: ∼7.2×10^11^ particles/mL). Silicon isolators composed of 12 wells (30 μL capacity) and targets (57 mm in diameter) purchased from Electron Microscopy Sciences.

### Methods

#### Crystallization and de-crystallization of uric acid on blank glass slides

The standard glass microscope slides were cut into eight equal pieces, cleaned and submerged in freshly prepared piranha solution (3:1 Sulfuric Acid: Hydrogen Peroxide) for 10 minutes, followed by thorough rinse with deionized water and air dry process. Silicon isolators (2.0 mm deep and 4.5 mm diameter) were attached to one piece of the cut glass slides. 20 μL uric acid solution (prepared by mixing 10 mg of uric acid with 20 mL of deionized water) were added to each wells and allowed to crystallize at room temperature.

After uric acid crystals were grown, 10 μL of bovine synovial fluid at room temperature (from Lampire Biological Laboratories) were added to the wells and four different experiments were carried out (Scheme 1-Bottom):

○ Experiment 1: uric acid crystals with gold colloids at room temperature, where 10 μL of gold colloids were incubated inside the wells at room temperature;
○ Experiment 2: uric acid crystals with gold colloids using microwave heating, where 10 μL of gold colloids were incubated inside the wells using microwave heating (700 W output kitchen microwave, power level 1);
○ Control 1: uric acid crystals without gold colloids at room temperature, where 10 μL of deionized water without gold colloids were incubated inside the wells at room temperature;
○ Control 2: uric acid crystals without gold colloids using microwave heating, 10 μL of deionized water without gold colloids were incubated inside the wells using microwave heating.

Optical images of the crystals were taken at one minute increments with an optical microscope to observe de-crystallization uric acid crystals (i.e., the samples are taken out of the microwave for 30 sec). The number and size of uric acid crystals were monitored using Motic software.

## Results and Discussion

**Fig. 1:**
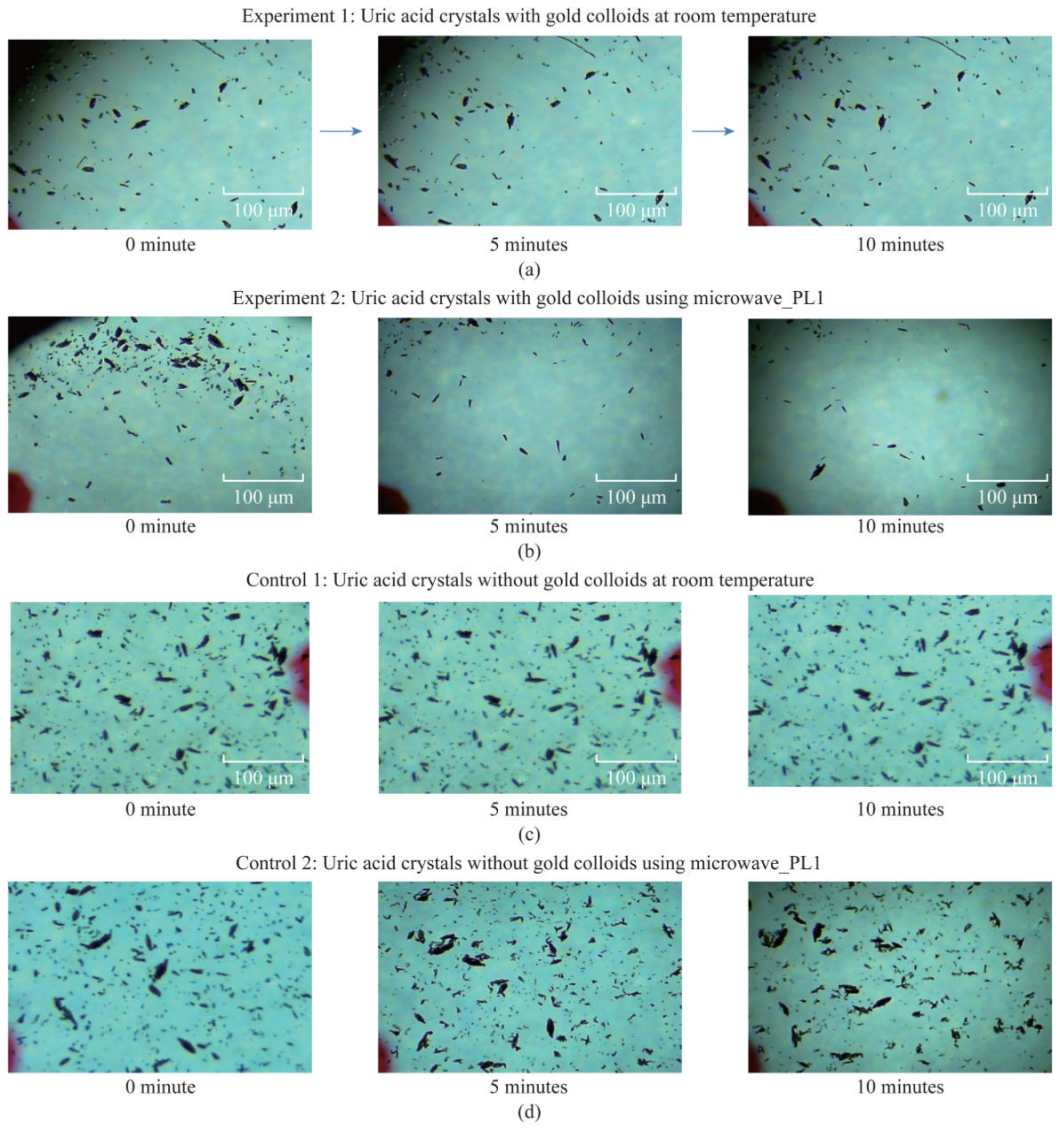
Optical images of uric acid crystals grown on glass slides with and without gold colloids at both room temperature and microwave heating after 10 minutes of incubation.

**Fig. 2:**
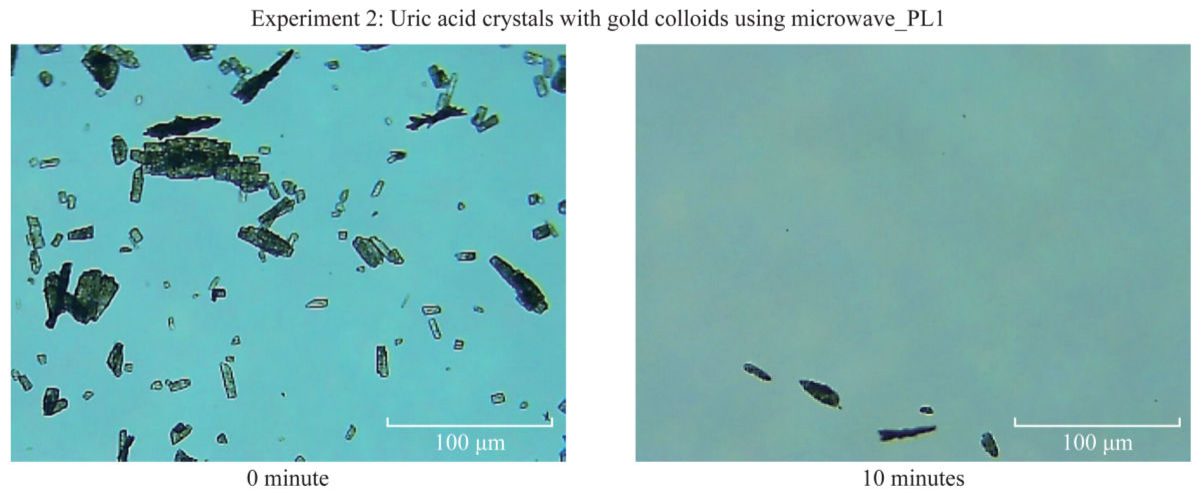
High-resolution optical images of uric acid crystals grown on glass slides with gold colloids and using microwave heating before and after 10 minutes of incubation.

Figure 1 shows the comparison of optical images of uric acid crystals with and without gold colloids incubated at room temperature and using microwave heating. In experiment 1 and Control 1, the size and the number of uric acid crystals remained the same after 10 minutes of incubation. The same observation was also made when gold colloids were incubated with uric acid crystals with microwave heating (Control 2). On the other hand, the use of gold colloids with microwave heating (Experiment 2) resulted in a significant reduction in the number and size of uric acid crystals after 10 minutes. Figure 2 shows the higher resolution optical images of uric acid crystals used in Experiment 2, which reveal that the initial size of the uric acid crystals (t=0 min) was 25±16 μm. The smaller uric acid crystals also appeared to be in an isolated form and aggregated form. However, after the addition of gold colloids and exposure to microwave heating, the number and size of the uric acid crystals were significantly reduced.

**Fig. 3:**
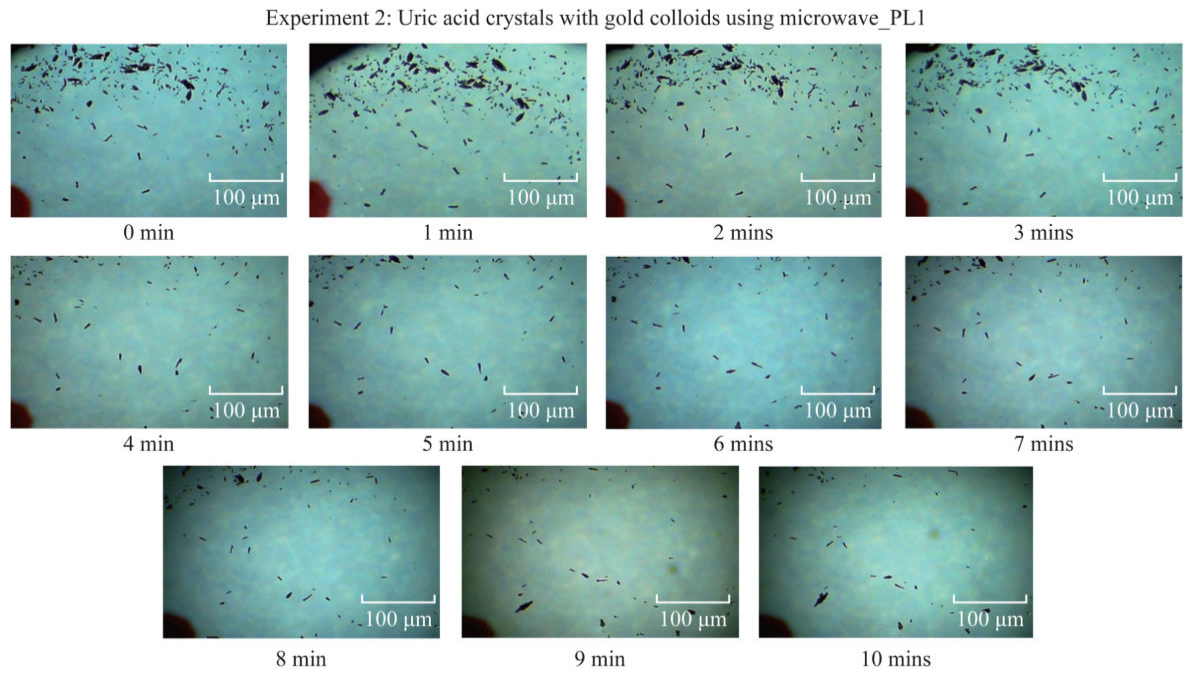
Time progression of the de-crystallization of uric acid crystals on blank glass slides with gold colloids using microwave heating at power level 1.

Figure 3 shows the time progression of uric acid crystals with the addition of gold colloids and microwave heating for 10 minutes. Figure 3 also shows that the number of uric acid crystals was significantly reduced after 4 minutes of microwave heating. The average number of uric acid crystals in the beginning of each experiment used in this study was 70±10.

**Fig. 4:**
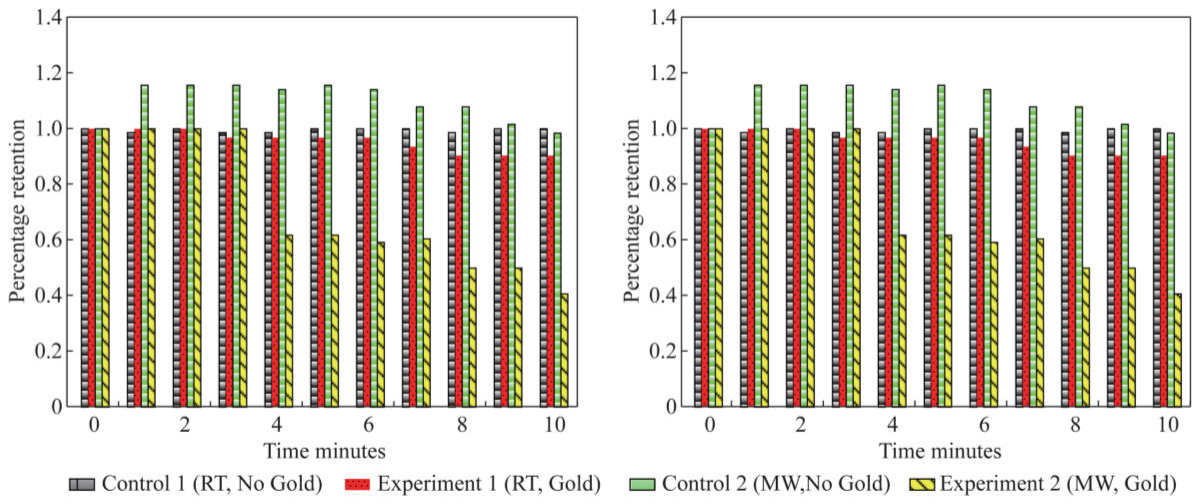
Calculated retention rate of uric acid crystals in all experiments.

In order to assess the effect of the use of gold colloids and microwave heating on uric acid crystals quantitatively, the percentage retention value of uric crystals in all experiments were calculated by dividing the number of crystals at any observation time by the initial number of uric acid crystals and shown on a scale 0 to 1 (Fig. 4). The percentage retention value of uric acid crystals without gold colloids at room temperature (Control 1) remained the same for 10 minutes, which indicated that uric acid crystals did not dissolve in synovial fluid. The incubation of uric acid crystals with the addition of gold colloids at room temperature (Experiment 1) showed a 5% decline in the percentage retention value, which implies that gold colloids in solution results in the de-crystallization of uric acid crystals. When uric acid crystals are exposed to microwave heating without gold colloids (Control 2), the percentage retention value is increased by 15% initially and decreased to the initial level thereafter, which can be attributed to the breakage of larger uric acid crystals into smaller ones (data not shown) and to the partial de-crystallization processes. When uric acid crystals are exposed to microwave heating after the addition of gold colloids (Experiment 2), the percentage retention value is decreased by 40% after 4 minutes and 60% after 10 minutes of microwave heating. The average number and size of uric acid crystals at the end of Experiment 2 was 30±5 and 19±10 micrometers, respectively.

It is important to comment on the mechanism behind microwave heating with gold colloids in the de-crystallization process and its comparison to de-crystallization at room temperature with gold colloids. The collision events between the gold colloids present in solution with the uric acid crystals on the glass surface are increased due to an increase in kinetic energy of gold colloids when exposed to microwave heating [9]. The collision events between gold colloids and uric acid crystals at room temperature are expected to be significantly less due the slow diffusion rates of gold colloids in solution [9] as compared to those exposed to microwave heating. In addition, the number of gold colloids (~10^12^ particles/mL, typical of chemically synthesized gold colloids) is significantly larger than the number of uric acid crystals (ca. 70 in this study), which results in greater collisions that breakdown and ultimately reduces the size of the uric acid crystals. We note that in our experiments the temperature of the synovial fluid was 24°C and we did not attempt to measure the temperature change during the exposure to microwave heating in this rapid communication. Based on our previous work, we can report that the temperature of the synovial fluid does not exceed 30°C after 1 minute of microwave heating at power level 1 (i.e., duty cycle of 3 sec) [7, 10]. The detailed investigation of de-crystallization of uric acid crystals is underway and will be reported in due course.

## Conclusions

In this work, we demonstrated the de-crystallization of uric acid crystals in the presence of gold colloids and low power microwave heating. In order to simulate the conditions in the human body, first, uric acid crystals were grown on glass slides (a model bone surface). Subsequently, gold colloids in synovial fluid was added to the glass slides with uric acid crystals and were exposed to microwave heating or incubated at room temperature. The exposure of uric acid crystals to microwave heating in the presence of gold colloids resulted in up to 60% reduction in the number of uric acid crystals. On the other hand, the use of colloids at room temperature, without microwave heating or no use of gold colloids did not affect the number and size of the uric acids crystals. The observations in this report demonstrate that the combined use of gold colloids and microwave heating can provide a framework for the further development of the proposed technique for future *in vivo* studies.
